# A computational scheme for internal models not requiring precise system parameters

**DOI:** 10.1371/journal.pone.0210616

**Published:** 2019-02-27

**Authors:** Dongwon Kim

**Affiliations:** 1 Department of Biongineering, School of Engineering, University of Maryland, College Park, MD, United States of America; 2 Department of Physical Therapy and Rehabilitation Science, School of Medicine, University of Maryland, Baltimore, MD, United States of America; Nanjing University, CHINA

## Abstract

Utilization by humans of a precise and adaptable internal model of the dynamics of the body in generating movements is a well-supported concept. The prevailing opinion is that such an internal model ceaselessly develops through long-term repetition and accumulation in the central nervous system (CNS). However, a long-term learning process would not be absolutely necessary for the formation of internal models. It is possible to estimate the dynamics of the system by using a motor command and its resulting output, instead of constructing a model of the dynamics with precise parameters. In this study, a computational model is proposed that uses a motor command and its corresponding output to estimate the dynamics of the system and it is examined whether the proposed model is capable of describing a series of empirical movements. The proposed model was found to be capable of describing humans’ fast movements which require compensation for system dynamics as well as sensory delays. In addition, the proposed model shows equifinality under inertial perturbations as seen in several experimental studies. This satisfactory reproducibility of the proposed computation raises the possibility that humans make a movement by estimating the system dynamics with a copy of motor command and sensory output on a momentary basis, without the need to identify precise system parameters.

## Introduction

Humans are endowed with a remarkable ability to execute limb movements even in the presence of changing loads arising either from interaction with the environment or from variation in properties of the sensorimotor system. Despite substantial delays in feedback loops and the dynamical properties of the sensorimotor system, they are capable of producing fast movements in a smooth trajectory. To explain these motor abilities, the so-called “internal model” was devised in the field of human motor behavior [1–10]. The internal model is a hypothesized controller residing in various brain regions including the motor cortex and/or cerebellum. Evidence of the existence of the internal model can be found in several phenomena. For instance, deafferented primates are able to reach a target point with their arms even in the absence of sensory feedback. Too, interacting forces between joints during movements need to be compensated to minimize movement error, yet this is nearly impossible with feedback control alone [4].

Though it is not a universal phenomenon, especially in destabilizing force fields [11–15], it is empirically observed that humans are capable of reaching a target position in a range of transient and smooth mechanical perturbations [16–21]. The equifinality property could not be explained using model-based motor control formulations with an internal model that suppose that the system adjusts the internal model by integrating the new load condition in response to the perturbation and accurately produces perfect corrective torques to bring the system to the same final position [21]. In model-based motor control formulations, integration of a perturbation into internal models requires practice involving considerable trials (e.g., [11]). However, equifinality is exhibited in movements without the need of adaptation to applied perturbations. Experimental evidence has supported equifinality by humans even within a single trial through online corrections. An intact monkey can return his arm back to a predefined trajectory immediately after his arm is perturbed while moving to a target [22]. Equifinality alludes the possibility that internal models can be formed and updated even on a momentary basis, as well as, on a trial-by-trial basis [11, 15, 23, 24]. This possibility is supported by research activities that have evaluated continuous adaptation of humans in motor behaviors [25–28].

An input-output relationship exists between the motor command and its resulting limb kinematics; the input acts on the neuromuscular system and environment in contact with the limb, while the output reflects the dynamic response. Although it is difficult to identify the properties of the system and environments with only the input and output available, the quantitative relationship between the input and output can be used to estimate the dynamics of the neuromuscular system for the purpose of formulating subsequent control actions. With an estimate of the limb and environment dynamics made at the previous step, the dynamics of the musculoskeletal system and environment in the current step could be compensated. In other words, the estimate can be used in place of a system model.

In fact, time-delay control (TDC) in the controls field utilizes the mechanism, which is called time-delay estimation (TDE) [29]. TDE uses the previous-step sensor reading and a record of previous-step command to quantitatively estimate the system dynamics and disturbances, which are otherwise difficult to identify precisely. The estimate cancels out the system dynamics and uncertainties through their incorporation into the control at the current step. Consequently, employing the TDE technique leads to accuracy and robustness of control in the presence of a wide class of uncertainties under infinitesimally small sampling intervals. TDE alleviates computation load for the system dynamics and uncertainties in comparison with other robust control schemes [30–34]. It does not require the use of high gain control. TDC provides an insight as to how humans form internal models.

In this study, a computational model of human motor control is proposed that estimates system dynamics in a similar way to TDC. The question as to whether humans estimate the dynamics of the neuromuscular system using the input-output relationship is examined. Regarding the issue of delays in sensory feedback loops, the proposed model handles these delays by taking the architecture of the Smith predictor. In 1993, Miall and colleagues borrowed the Smith predictor from control engineering to describe behaviors of biological systems with delays in feedback loops [35]. The Smith predictor explained how biological systems might overcome feedback delays. The Smith predictor is a model-based controller, and accordingly requires system parameters. The forward and inverse models of the Smith predictor are liberated from the requirement for a model of the dynamics with precise parameters with the aid of the TDC principle.

Through simulation studies, it is investigated whether or not the proposed computation is capable of reproducing a series of fast movements. Feedback control alone cannot be considered for fast movements due to the delays inherent in sensory systems. Anticipatory control using forward models needs to be involved to make an appropriate action before sensory information is available. Also, feedback control alone cannot compensate for intersegmental interaction forces that arise during multi-joint movements [4]. These movements support the existence of internal models. If the proposed computation successfully reproduces fast movements, this would imply the possibility that the human motor control system produces movements in a similar manner to the operation of TDC. That is, in humans it would be possible to estimate system dynamics using the input-output relationship, not requiring a model of limb dynamics with precise parameters. This hypothesis is in line with the perspective that a functionally good enough representation/estimation of the system is sufficient to produce a rapid response to perturbations [36, 37].

A further investigation regarding whether the proposed computation captures movements under unexpected change in load is carried out. Perturbations by inertial changes would offer a tool to investigate the issue regarding whether or not internal models involve a precise system model. Attempts to reproduce the unexpectedly perturbed movements with a model-based internal model controller and the proposed controller provide an insight into the possibility that humans make movements without system parameter identification.

## Computational model development

### Computed torque method for human movements

In 2002, Jagacinski and Flash introduced a controller to describe human movements [38]. This controller, which is based on the computed-torque control (CTC) for a single-DOF arm model, is re-formulated here for a multi-DOF arm model.

The dynamics of the arm is generally described as

$$\begin{array}{c} M\left(\theta \right)\ddot{\theta }+V\left(\theta ,\dot{\theta }\right)+G_{r}\left(\theta \right)+F\left(\dot{\theta }\right)+U=\tau , \end{array}$$ (1)

where *θ* ∈ *R*^*n*^ denotes the joint angles; *M*(*θ*) ∈ *R*^*n*×*n*^ the inertia matrix; $V\left(\theta ,\dot{\theta }\right)\in R^{n}$ the Coriolis and centrifugal forces; *G*_*r*_(*θ*) ∈ *R*^*n*^ the gravitational forces; $F\left(\dot{\theta }\right)\in R^{n}$ the friction; *U* the unknown disturbances; *τ* ∈ *R*^*n*^ the control inputs.

Eq (1) is considered as a system model throughout this study. The controller presented in [38] for the arm model consists of system dynamics cancellation, the feedforward component and the feedback component.

$$\begin{array}{c} \tau =\underset{\text{Dynamics}\;\text{cancellation}}{\underset{︸}{V\left(\theta ,\dot{\theta }\right)+G_{r}\left(\theta \right)+F\left(\dot{\theta }\right)+U}}+\underset{\text{Feedforward}}{\underset{︸}{M\left(\theta \right){\ddot{\theta }}_{d}}}+\underset{\text{Feedback}}{\underset{︸}{K_{v}\left({\dot{\theta }}_{d}-\dot{\theta }\right)+K_{p}\left(\theta_{d}-\theta \right)}}, \end{array}$$ (2)

where *θ*_*d*_ ∈ *R*^*n*^ denotes a desired angle vector, and *K*_*v*_, *K*_*p*_ ∈ *R*^*n*×*n*^ are diagonal viscosity and stiffness matrices with diagonal elements *K*_*v*1_, *K*_*v*2_, ⋯, *K*_*vn*_ and *K*_*p*1_, *K*_*p*2_, ⋯, *K*_*pn*_, respectively.

Dynamics cancellation is carried out by putting force components of the arm system into the system through the control inputs. The feedforward component is proportional to the desired acceleration ${\ddot{\theta }}_{d}$, because the controlled system is an acceleration control system [38]. The feedforward control utilizes the inverse dynamics of the system; the torque component is created by the acceleration of the desired trajectory, which is programmed according to an intended movement. In the case that the initial condition of the arm is quiescent and no uncertainty exists, the actual position of the arm converges to the desired one [38]. The third component, feedback, plays a role in diminishing the error between the desired trajectory and actual trajectory measured by the sensory systems.

Injecting the control inputs into the arm produces the following error dynamics:

$$\begin{array}{c} 0=M\left(\theta \right)\left({\ddot{\theta }}_{d}-\ddot{\theta }\right)+K_{v}\left({\dot{\theta }}_{d}-\dot{\theta }\right)+K_{p}\left(\theta_{d}-\theta \right). \end{array}$$ (3)

**Fig 1:**
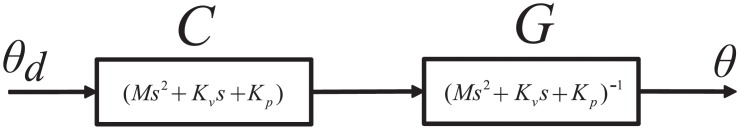
Simplified block diagram of the arm system linearized by the CTC. The closed-loop dynamics of the arm can be expressed as a cascade combination of a controller *C* and a plant *G*. *θ*_*d*_ and *θ* denote the desired angle and actual angle, respectively.

From this error dynanmics, a simplified block diagram of the arm system linearized by the controller can be derived as shown in Fig 1. The closed-loop dynamics of the arm can be expressed as a cascade combination of a controller *C* and a plant *G*.

However, the CTC is not tolerant to sensory delays. This indicates that the CTC is not suitable to describe human motor control involving sensory delays.

### Smith predictor

**Fig 2:**
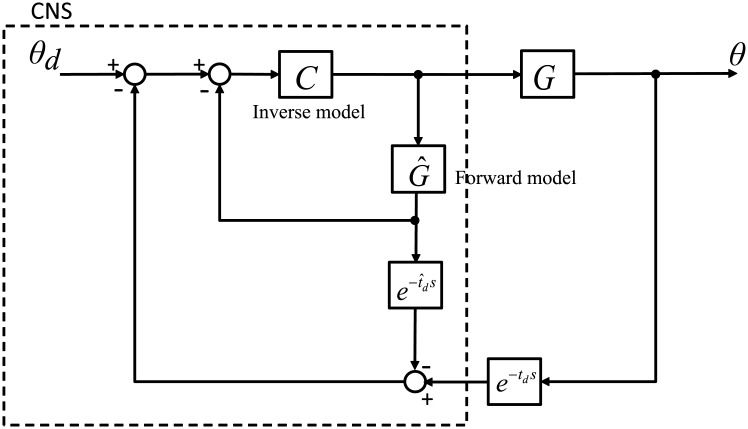
Smith predictor. The Smith predictor is a model-based controller developed for processes with a long time delay in feedback loops. This control intentionally delays the estimated state as long as the actual state is delayed so that the delayed actual state and delayed estimated state cancel one another.

The Smith predictor, proposed in [39], is a control architecture for systems with delays, as shown in Fig 2. The outer control loop feeds back the actual state of the system *G*, but due to the delay of the feedback loop, use of the outer loop alone would not provide satisfactory control performance and lead to instability in the worst case. Thus, the inner loop is added to send the (estimated) current state to the controller *C*. The current state is estimated using a system model $\hat{G}$ that is supposed to be simulated using a copy of the control input. The Smith predictor delays the estimated state as long as the actual state is delayed so that the delayed actual state and delayed estimated state cancel one another. If the perfect match between these two delayed states is made, the controller *C* can show control performance with no influence of delay on the outer feedback loop. Miall and colleagues [35] employed this Smith predictor in human control modelling to describe motor ability by the CNS even in the face of sensory delays. They assumed that the controller *C* and system model $\hat{G}$ are devised for the inverse model and forward model in the cerebellum, respectively.

Now, let us try to combine the CTC with the Smith predictor. From the lineaized arm system with the CTC, which is shown in Fig 1. The controller *C*_*CTC*_ of the CTC can match with the controller *C* of the Smith predictor and the linearized plant *G*_*CTC*_ of the CTC with the plant *G* of the Smith predictor. Then the forward model can be expressed as

$$\begin{array}{c} \hat{G}\left(s\right)={\left(Ms^{2}I+K_{v}s+K_{p}\right)}^{-1}. \end{array}$$ (4)

**Fig 3:**
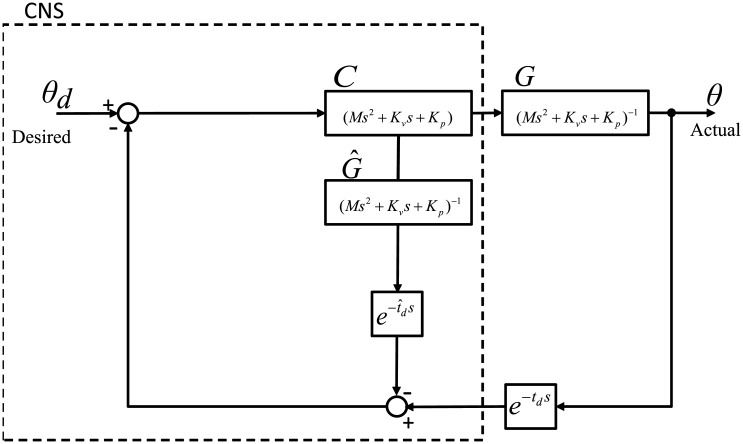
Simplified block diagram of the CTC in the architecture of the Smith predictor. The controller in this control architecture can match with the controller *C* of the Smith predictor and the linearized plant of the CTC with the plant *G* of the Smith predictor. The forward model equals to the plant linearized by the CTC. This control architecture compares the actual position that is delayed in feedback loop with its estimate that is intentionally delayed. The difference between them is compared with the desired position.

Note that the forward model equals to the plant linearized by the CTC. This implies that the forward model provides estimated states using the same plant dynamics controlled under the CTC. Fig 3 displays a simplified block diagram of the CTC in the architecture of the Smith predictor.

### Proposed control

So far, the CTC has been combined with the Smith predictor to liberate the CTC from the sensory delay issue. But the CTC does require a model of the dynamics with precise parameters. Time-delay estimation (TDE) can eliminate this requirement while it reduces computational load.

The equations of motion (1) can be algebraically manipulated to express it with an explicit input-output relationship. Introducing a diagonal constant matrix $\bar{\beta }\in R^{n\times n}$ consisting of diagonal elements ${\bar{\beta }}_{1},{\bar{\beta }}_{2},\cdots ,{\bar{\beta }}_{n}$, and grouping the system dynamics plus the disturbance term *U* into one term (*H*), Eq (1) is expressed as follows:

$$\begin{array}{l} \;\bar{\beta }{\ddot{\theta }}_{\left(t\right)}-\bar{\beta }{\ddot{\theta }}_{\left(t\right)}+M\left(\theta_{\left(t\right)}\right){\ddot{\theta }}_{\left(t\right)}+V\left(\theta_{\left(t\right)},{\dot{\theta }}_{\left(t\right)}\right)+G\left(\theta_{\left(t\right)}\right)+F\left({\dot{\theta }}_{\left(t\right)}\right)+U_{\left(t\right)}\\ =\bar{\beta }{\ddot{\theta }}_{\left(t\right)}+H_{\left(t\right)}\\ =\tau_{\left(t\right)}, \end{array}$$ (5)

where

$$\begin{array}{c} H_{\left(t\right)}≜-\bar{\beta }{\ddot{\theta }}_{\left(t\right)}+M\left(\theta_{\left(t\right)}\right){\ddot{\theta }}_{\left(t\right)}+V\left(\theta_{\left(t\right)},{\dot{\theta }}_{\left(t\right)}\right)+G\left(\theta_{\left(t\right)}\right)+F\left({\dot{\theta }}_{\left(t\right)}\right)+U_{\left(t\right)}. \end{array}$$ (6)

In Eq (5), the input *τ* is linked to the output $\ddot{\theta }$ through the term *H*, which suggests that the term *H* can be quantitatively estimated using the input and output. Meanwhile, it is acceptable to assume that the term *H* is piece-wise continuous if the disturbance term *U* is continuous. This implies that the value of the term *H*_(*t*)_ at an instant *t* can be approximated by its value at the previous instant *t* − *dt*. The shorter the gap *dt* between two consecutive instants of time leads to the more accurate approximation. An estimate of the value of the term *H* can be obtained in this way:

$$\begin{array}{c} H_{\left(t\right)}\approx H_{\left(t-dt\right)},\;{\hat{H}}_{\left(t\right)}=H_{\left(t-dt\right)}. \end{array}$$ (7)

In practice, the value of the *H* at the previous instant can be calculated using the input and output at the previous instant, as suggested in Eq (5), as follows:

$$\begin{array}{c} H_{\left(t-dt\right)}=\tau_{\left(t-dt\right)}-\bar{\beta }{\ddot{\theta }}_{\left(t-dt\right)}. \end{array}$$ (8)

If the value of ($\tau_{\left(t-dt\right)}-\bar{\beta }{\ddot{\theta }}_{\left(t-dt\right)}$) is included in the motor command, the system dynamics plus the disturbance *U* are cancelled out. As in the CTC, the motor command injects feedforward torques (${\ddot{\theta }}_{d}$) and restoring torques that can be realized by placing a spring (*K*_*p*_) and damper (*K*_*v*_) between the actual limb position and the desired limb position of each joint.

Then, the control law is expressed as

$$\begin{array}{c} \tau_{\left(t\right)}=\tau_{\left(t-dt\right)}-\bar{\beta }{\ddot{\theta }}_{\left(t-dt\right)}+\bar{\beta }({\ddot{\theta }}_{d}{}_{\left(t\right)}+K_{v}\left({\dot{\theta }}_{d}{}_{\left(t\right)}-{\dot{\theta }}_{\left(t\right)}\right)+K_{p}\left(\theta_{d}{}_{\left(t\right)}-\theta_{\left(t\right)}\right)). \end{array}$$ (9)

TDC provides the similar desired error dynamics as the CTC:

$$\begin{array}{c} 0={\ddot{\theta }}_{d}-\ddot{\theta }+K_{v}\left({\dot{\theta }}_{d}-\dot{\theta }\right)+K_{p}\left(\theta_{d}-\theta \right). \end{array}$$ (10)

Accordingly, TDC can be adjustable to the architecture of the Smith predictor with a forward model, which is selected as

$$\begin{array}{c} \hat{G}\left(s\right)={\left(s^{2}I+K_{v}s+K_{p}\right)}^{-1}. \end{array}$$ (11)

**Fig 4:**
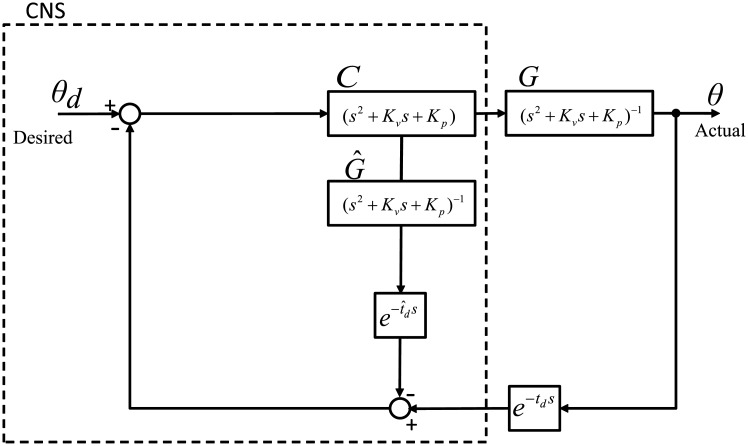
Simplified block diagram of TDC in the architecture of the Smith predictor. The controller in this control architecture can match with the controller *C* of the Smith predictor and the linearized plant with the plant *G* of the Smith predictor. The forward model equals to the plant linearized by this control.

Fig 4 displays a simplified block diagram of TDC in the architecture of the Smith predictor. Now, let us turn our attention to how delayed estimates can be obtained from the forward model.

From the error dynamics (10), it is possible to obtain the estimates of the state vectors $\theta ,\dot{\theta }$ and $\ddot{\theta }$, defined as $\hat{\theta },\dot{\hat{\theta }}$ and $\ddot{\hat{\theta }}$ respectively, as follows:

$$\begin{array}{c} {}[\begin{array}{c} \hat{\theta }\\ \dot{\hat{\theta }} \end{array}]=e^{At}[\begin{array}{c} {\hat{\theta }}_{0}\\ {\dot{\hat{\theta }}}_{0} \end{array}]+\int_{0}^{t}e^{At^{\prime }}[\begin{array}{c} 0\\ I \end{array}]p\left(t-t^{\prime }\right)dt^{\prime }, \end{array}$$ (12)

$$\begin{array}{c} \ddot{\hat{\theta }}=-K_{v}\dot{\hat{\theta }}-K_{p}\hat{\theta }+p, \end{array}$$ (13)

where

$$\begin{array}{c} A≜[\begin{array}{cc} 0 & I\\ -K_{p} & -K_{v} \end{array}], \end{array}$$ (14)

$$\begin{array}{c} p≜{\ddot{\theta }}_{d}+K_{v}{\dot{\theta }}_{d}+K_{p}\theta_{d}. \end{array}$$ (15)

The initial values of estimates $\hat{\theta },\dot{\hat{\theta }}$ can be assumed to be the same as those of the actual state vectors $\theta ,\dot{\theta }$.

With the forward model, the current states are estimated and fed back to the controller. Note that this forward model does not require a model of the dynamics with precise parameters.

As shown in Fig 4, the actual position that is delayed in the feedback loop and its estimate that is intentionally delayed are compared. The difference between them is compared with the desired position. These differences can be expressed as *θ*_*r*_:

$$\begin{array}{c} \theta_{r(t)}≜\theta_{d(t)}-\left(\theta_{\left(t-t_{d}\right)}-{\hat{\theta }}_{\left(t-{\hat{t}}_{d}\right)}\right), \end{array}$$ (16)

where *t*_*d*_ denotes the time delay of the feedback loop and ${\hat{t}}_{d}$ is its estimate.

Then, the controller forces the controlled system to follow *θ*_*r*_, reflecting that the controller receives the current state estimated by the forward model. The control law of the proposed model is designed as

$$\begin{array}{c} \tau_{\left(t\right)}=\tau_{\left(t-dt\right)}-\bar{\beta }{\ddot{\hat{\theta }}}_{\left(t-dt\right)}+\bar{\beta }({\ddot{\theta }}_{r(t)}+K_{v}\left({\dot{\theta }}_{r(t)}-{\dot{\hat{\theta }}}_{\left(t\right)}\right)+K_{p}\left(\theta_{r(t)}-{\hat{\theta }}_{\left(t\right)}\right)). \end{array}$$ (17)

According to the closed-loop dynamics that the controller pursues, one of the formulations for the forward model needs to be modified to

$$\begin{array}{c} p={\ddot{\theta }}_{r}+K_{v}{\dot{\theta }}_{r}+K_{p}\theta_{r}. \end{array}$$ (18)

It would be possible to assume that the CNS selects appropriate values of the matrix $\bar{\beta }$ and modulates the muscle viscosity and stiffness *K*_*v*_, *K*_*p*_ according to a given task. The matrix $\bar{\beta }$ determines the accuracy of dynamics estimation by TDE. A proof is presented in an Appendix.

## Simulation

The proposed control model is validated through a series of simulation studies, examining whether the model is capable of reproducing empirical phenomena obtained from human subject experiments.

The first study investigates whether or not the proposed model is able to reproduce fast movements of short duration during which the sensory delay is too long to allow feedback corrections. It is predicted that computational models with anticipatory control can reproduce fast movements.

The second study evaluates whether the proposed model is capable of dealing with interaction forces that arise from two-joint movements. The proposed model is predicted to compensate for interaction forces successfully.

The last study attempts to address the issue regarding whether or not internal models are formed grounded in a model of the dynamics of the system. Model-based computation presents prediction that varies with changes in the dynamic system. If a load unexpectedly perturbs the system during movement, the system output would deviate from the planned end-point. In contrast, the proposed computational model, which is not model-based, would show that the planned end-point is reached even in the presence of a perturbation.

### Arm model implementation

**Fig 5:**
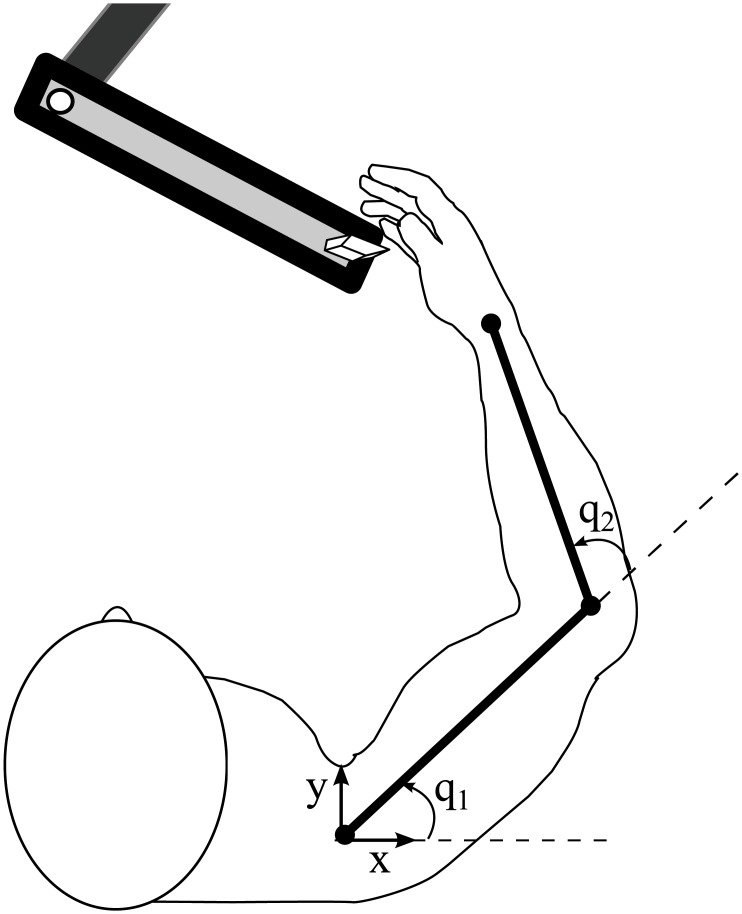
Schematic of a planar reaching experiment. The arm is modeled with a 2 degree-of-freedom (DOF) system involving the shoulder and elbow joints.

The experimental data considered in this study were produced by horizontal arm movements; the gravitational force was neglected. The arm is modeled with a 2 DOF system involving the shoulder and elbow joints, as depicted in Fig 5 and described as follows:

$$\begin{array}{c} M(q)\ddot{q}+C(q,\dot{q})=\tau , \end{array}$$ (19)

where

$$\begin{array}{c} M(q)≜[\begin{array}{cc} M_{11} & M_{12}\\ M_{21} & M_{22} \end{array}], \end{array}$$ (20)

$$\begin{array}{c} M_{11}≜J_{1}+J_{2}+M_{1}l_{m1}^{2}+M_{2}\left(l_{1}^{2}+l_{m2}^{2}+2l_{1}l_{m2}\text{cos}q_{2}\right), \end{array}$$ (21)

$$\begin{array}{c} M_{12}=M_{21}≜J_{2}+M_{2}\left(l_{m2}^{2}+l_{1}l_{m2}\text{cos}q_{2}\right), \end{array}$$ (22)

$$\begin{array}{c} M_{22}≜J_{2}+M_{2}l_{m2}^{2}, \end{array}$$

and

$$\begin{array}{c} C(q,\dot{q})≜[\begin{array}{c} M_{2}l_{1}l_{m2}\text{sin}q_{2}\left(2{\dot{q}}_{1}+{\dot{q}}_{2}\right){\dot{q}}_{2}\\ M_{2}l_{1}l_{m2}\text{sin}q_{2}{\dot{q}}_{1}^{2} \end{array}]. \end{array}$$ (23)

The parameters of the arm are adopted from [24]; for the upper arm, *J*_1_ = 0.0141 (kgm^2^), *M*_1_ = 1.93 (kg), *l*_1_ = 0.31 (m), *l*_*m*1_ = 0.165 (m), for the forearm, *J*_2_ = 0.0188 (kgm^2^), *M*_2_ = 1.52 (kg), *l*_2_ = 0.34 (m), *l*_*m*2_ = 0.19 (m). The lengths *l*_*m*1_ and *l*_*m*2_ denote the distances between the center of mass and the proximal joint.

It is assumed that human subjects plan a minimal-jerk trajectory in joint space and Cartesian space according to the given task [40]. The desired (planned) trajectory is designed as

$$\begin{array}{c} q_{d(t)}=q_{0}+\left(q_{f}-q_{0}\right)\left(6t^{\prime 6}-15t^{\prime 5}+10t^{\prime 4}\right),\;t^{\prime }=\frac{t}{T}, \end{array}$$ (24)

where *q*_0_, *q*_*f*_ denote the initial and final positions of the hand, *T* denotes the duration of the movement.

### Movements to be reproduced

#### I. Single-joint fast movement

Kistemaker and colleagues investigated single-joint (elbow) fast movements [41]. Participants were asked to direct their hand from the marked intermediate of one block to that of another block as fast as possible, once an auditory cue was presented. The participants practiced until they could move quickly to the target with minimal overshoot. Fig 4A in [41] shows trajectories by 6 participants of a flexion of 145 degrees starting from an initial flexion of 45 degrees for a duration of 0.2 s. Note that these trajectories can be described by the minimum-jerk trajectory.

#### II. Two-joint fast movement

Koike and colleagues had a participant make five movements to five different positions with a duration between 0.5 s and 0.75 s using the shoulder and elbow joints [42]. It was observed that deviations from the desired paths were more significant for fast movements than those for slow movements since fast movements involve larger interaction forces. Also it was found that deviations from transverse paths were more significant than those from radial paths. The path from point (-0.2 m, 0.5 m) to point (0.25 m, 0.35 m) is selected for this simulation study. A comparison in movements between 0.5 s and 1 s is made to examine whether the proposed control is able to compensate for increased interaction forces during fast movements. The desired paths are designed by the minimum-jerk trajectory as in [4].

#### III. Movement with unexpected inertial changes

Pinter and colleagues studied kinematic changes during movements under unexpected inertial perturbations [43]. They asked participants to move their elbow joint from a 50 degree to an 85 degree of flexion angle as fast as possible within 0.2 s but the inertial load was changed without informing the participants. The participants familiarized themselves with movements with an inertial load that was then replaced with either a lighter one (25% decrease) or a heavier one (25% increase).

The main findings of the study are that there is no significant error in the end-point position in the presence of changes in inertial loads and that motor commands are customized for a certain inertial load taking several trials.

In the present study, the trial of the low inertial load following continuous trials of the intermediate inertial load is labelled ML and the trial of the high inertial load following continuous trials of the intermediate inertial load is labelled MH. The label MM is made in a similar way.

### Computational models for comparison

For a comparative study with a model-based control system, an optimal control model is chosen which supports the internal-model hypothesis [44–47]. This model is accompanied by a predictor for estimating the current states based on the sensory information delayed on feedback loops. Details on the optimal control used in this study are presented in the Appendix.

The other computational model is an equilibrium-point controller that operates using sensory information with no anticipatory component. A modified equilibrium-point controller that was developed by adding velocity feedback loop to accommodate feedback delays is adopted [48, 49]. The control law is

$$\begin{array}{c} \tau =D\left({\dot{\theta }}_{d(t)}-{\dot{\theta }}_{\left(t-t_{dv}\right)}\right)+P\left(\theta_{d(t)}-\theta_{\left(t-t_{dp}\right)}\right), \end{array}$$ (25)

where *D*, *P* are feedback gains and *t*_*dv*_, *t*_*dp*_ denote feedback delays on velocity loop and position loop.

### Simulation settings

Feedback signals are considered as an integration of all usable sources of sensory information about limb movements including proprioceptive and visual feedback. The sensory delay *t*_*d*_ is moderately set as 65 ms [4, 24]. Since a perturbation in sensory feedback was not given in the experiment, it is assumed that the CNS estimates the exact value of the sensory delay so ${\hat{t}}_{d}$ is set to 65 ms. For the equilibrium-point model, a delay of 65 ms is put into the position feedback loop while a delay of 25 ms is imposed on the velocity feedback loop.

For the study involving fast movements, all control gains are tuned so that they, within a reasonable range, give the minimal deviation from the desired trajectory between 0.1 s and 0.3 s (see Fig 4A in [41]). After the 0.3 s, it would be possible that the arm impedance drastically changes to stop the arm movement at the planned position. This range is not taken into account in the simulation studies where impedance is assumed to be constant. In the case of the optimal control, a steady-state linear quadratic (LQ) method based on the solution of the algebraic Riccati equation is used to set the values of the matrix *K*.

For the study about movements under unexpected inertial load changes, it is the first task to ascertain control gains that enable the arm to closely follow the minimal-jerk trajectory between the angles of 50 and 85 degrees for 0.25 s, under the intermediate load (*J* = 0.165 kgm^2^). Then, the load is replaced with either a lighter load (*J* = 0.12) or a heavier load (*J* = 0.205), while the control gains remain the same. In the case of the optimal control, the predictor (41) estimates the current states with *J* = 0.165 (the CNS expects the intermediate inertial load). The LQ method is used to determine the values of the matrix *K*.

Simulations are conducted in Matlab using ODE45. The optimization toolbox in Matlab is used to check out the possibility of a significantly improved fit between the simulated trajectory and empirically observed trajectory.

## Results

### Single-joint fast movement

**Fig 6:**
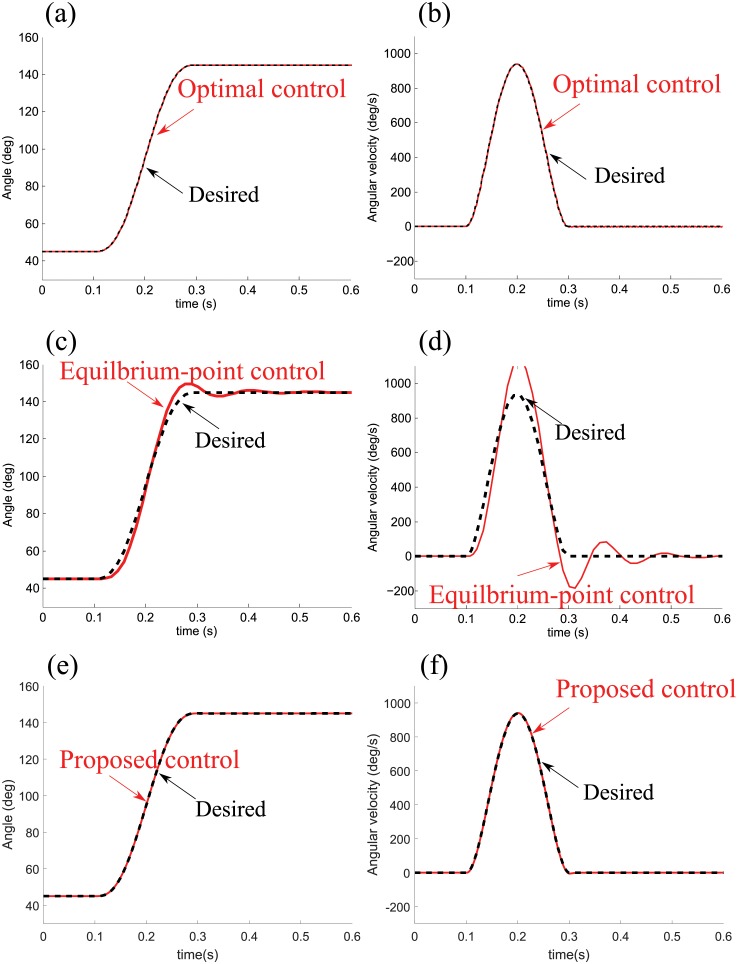
Simulated fast movement with a delay of 65 ms. A single-joint fast movement are reproduced by the optimal control, equilibrium-point control and proposed control. The optimal control (a, b) and proposed control (e, f) reproduce the minimum-jerk trajectory, while the equilibrium-point control (c, d) fails to reproduce the minimum-jerk movement within the range between the 0.1 s and 0.3 s.

Fig 6 presents the simulation results of the single-joint fast movement by the optimal control (model-based internal model control), equilibrium-point control and proposed control. The outputs of the optimal control model follow the minimum-jerk trajectory. The optimal control is supposedly provided with the exact parameters of the system since the experimental results Fig 3 are derived from sufficiently practiced movements.

The values of *Q* and *R* in Eq (40) are set to [10 0;0 10] and 2, respectively, but as long as values of *Q* and *R* are not too low, it is observed that the optimal control can produce the minimum-jerk trajectory, noting that the forward model for this computation effectively deals with the delay of 65 ms on the feedback loop (41).

For the equilibrium-point control, the P gain and D gain in (25) are tuned to 0 and 0.76, respectively (the velocity feedback loop is faster than the position feedback loop). Note that the control gains are tuned by the optimization toolbox until the best match with the planed trajectory is achieved. However, this computation is unable to reproduce the minimum-jerk movement. This result implies that the non-existence of the forward model leads to the failure of reproducing.

The proposed control reproduces the minimum-jerk movement within the range between the 0.1 s and 0.3 s. The values of *K*_*v*1_ and *K*_*p*1_ (1 DOF) are selected as 200 and 500, respectively. The value of the gain ${\bar{\beta }}_{1}$ (1 DOF) is arbitrarily set to 0.01. Too low and too high values of ${\bar{\beta }}_{1}$ result in deviations from the minimum-jerk trajectory or instability. The successful reproduction of the experimental behavior implies that the forward model works in the presence of the sensory feedback delay.

**Fig 7:**
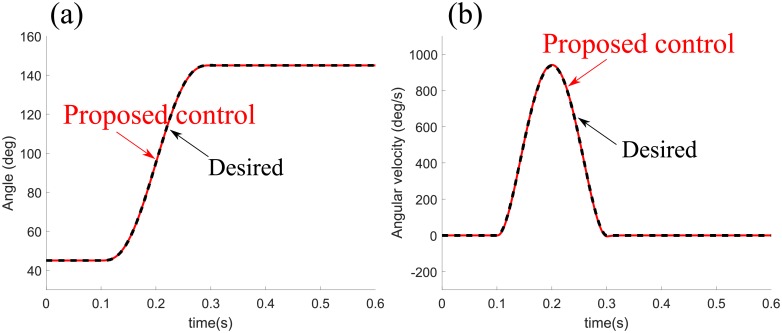
Simulated fast movement with a delay of 300 ms. The position and velocity profiles of a fast movement are produced under a delay of 300 ms, with the gain values remaining the same as the fast movement under a delay of 65 ms. The proposed control follows the minimum-jerk trajectory with the increased delay.

Fig 7 shows the position and velocity profiles of a fast movement by the proposed control under a delay of 300 ms, with the gain values remaining the same. The proposed control tends to follow the minimum-jerk trajectory with the increased delay.

### Two-joint fast movement

**Fig 8:**
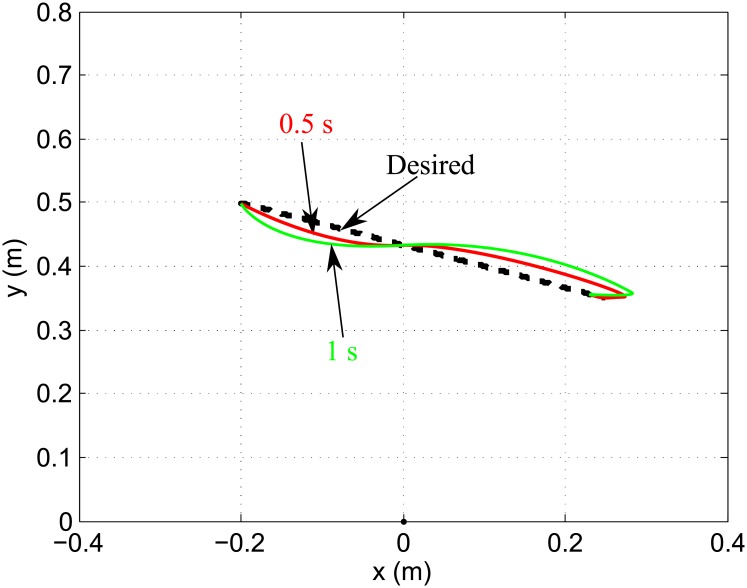
Simulated movement of a 2-joint planar arm with a duration of 0.5 s and 1 s. A linear movement with a duration between 0.5 s and 0.75 s using the shoulder and elbow joints is reproduced by the proposed control. A faster movement involves larger interaction forces. A comparable deviation is produced by the proposed control for both fast and slow movements, indicating that the proposed control successfully compensates for the interaction forces between the two joints.

Fig 8 shows simulated movements of a 2-joint planar arm by the proposed control, with the duration varying from 0.5 s to 1 s. A comparable deviation is produced by the proposed control for both fast and slow movements. This suggests that the proposed control successfully compensates for the interaction forces between the two joints.

In the proposed control, the matrix $\bar{\beta }$ is selected as [0.018 0;0 0.015] (2 DOF). The matrices *K*_*v*_, *K*_*p*_ are set to be [25 0;0 25] and [150 0;0 150], respectively.

### Movement with unexpected inertial changes

**Fig 9:**
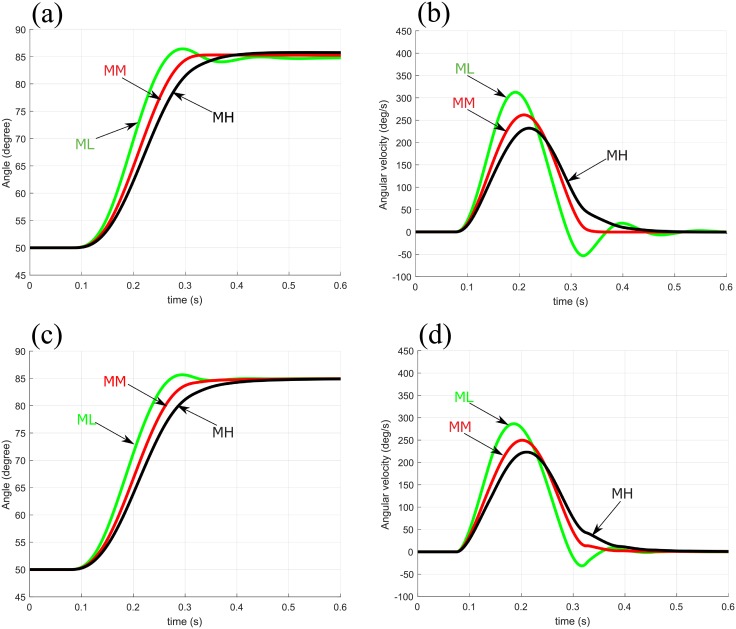
Simulated movements. The position and velocity are exhibited on the left and right panels, respectively, under unexpected inertial load changes with the optimal control (a, b) and the proposed control (c, d). ML stands for the inertial change from the intermediate load to the low load; MH from the intermediate load to the high load. MM indicates no change from the intermediate load. The output of the proposed model converges to the target position regardless of inertial loads presented, whereas the optimal model does not; the end-point errors of the optimal model are notably higher than those of the proposed model. Since the proposed model eliminates the system dynamics and injects planned dynamics, it is reasonable to expect the system simulated by the proposed control converges to a desired point.

Fig 9 exhibits predictions of the effect of unexpected load changes on elbow flexion movements by the optimal control and proposed control models. The predictions show the plots of the elbow angle and its velocity in the cases that the low, intermediate and high inertial loads are presented following consecutive trials with the intermediate load. For the optimal control, the values of *Q* and *R* in (40) are selected as [500 0;0 500]^*T*^ and 2, respectively, which allow the system to follow the minimum-jerk trajectory for the intermediate load condition. For the proposed control, the values of *K*_*v*1_ and *K*_*p*1_ (1 DOF) are selected as 25 and 150, respectively. The value of the gain ${\bar{\beta }}_{1}$ (1 DOF) is arbitrarily selected as 0.15 to follow a minimum-jerk trajectory under the intermediate load condition.

It is observed that the two models reproduce the empirical results with the same patterns in terms of the peak angular velocity, the time to peak angular velocity relative to the total movement time, and the number of oscillations from the reversal point reversal point at which absolute angular velocity dropped below 5 degrees/s to the point where the amplitude of the oscillations drops below 2 degrees. All three measures show a decrease as the inertial load unexpectedly presented increases (please refer to Fig 2 in [43]).

However, the two computational models offer different predictions in terms of the distance between the target point and reversal point. While the output of the proposed model converges to the target position regardless of inertial loads presented, the optimal model does not. The two trajectories of conditions ML and MH in the optimal prediction reach the vicinity of the target point, but the end-point errors of the optimal model are notably greater than those of the proposed model, although the optimal model generates greater amplitude commands than the proposed model. Since the proposed model eliminates the system dynamics and injects planned dynamics, it is reasonable to expect the system simulated by the proposed control converges to a desired point asymptotically. Meanwhile, in the optimal control, estimates of the current state are produced with a system model in forward models and are fed back to internal models. If the system model becomes inaccurate due to unexpected loads, then the estimates become inaccurate accordingly, which leads to residual error in the end-point.

## Discussion

TDC-type control shows robust performance against system parameter uncertainties. Using the motor command and its outcome at the previous instant, TDC-type control estimates the controlled system dynamics interacting with the environment. With no need of high stiffness and damping gains, TDC-type control achieves accurate and robust tracking tasks. Humans show a remarkable ability to execute limb movements even in the presence of changes in the environment as well as in the properties of the sensorimotor system, even with low stiffness [50]. Even in the case that the arm is perturbed gradually or abruptly during task, corrective torques will be generated to compensate for the perturbation and the arm will be positioned as planned in the end. These common points bring forward a question regarding whether humans control their limbs in a similar way to TDC-type control. This study proposed a computational scheme of human control based on the TDC principle and evaluated the possibility that the model captures empirical phenomena.

### Model of human control

The proposed computation consists of the inverse model and forward model components as in typical computational models that support the existence of internal models in the CNS [2, 3, 6, 35, 45, 51–53]. From the view of advocators for the role of internal models in motor control, when initiating a reach to a target, a human sets a motor plan including a trajectory to be followed, based on the initial movement conditions. During movement, a forward model of the biological system integrates sensory inflow and a copy of motor outflow to estimate the consequence of the motor commands sent to a limb [1, 54–56]. The estimated position of the movement is continuously compared to the target position and the differences between them cause an error signal that modifies the motor command. The proposed model follows this mechanism, except for some points presented below.

The inverse model of the proposed computation calculates motor command that moves the controlled limb to a desired state. The inputs to the inverse model include an efferent copy of motor command and the position, velocity and acceleration values of the desired limb state and (estimated) actual limb state. Unlike typical internal-model-based controllers, an efferent copy of the motor command is sent to inverse models as well as forward models. The copy, with its estimated corresponding acceleration of the limb, is used to cancel out the dynamics of the limb and environment, and desired dynamics injected is formed by a combination of the position, velocity and acceleration of the limb based on muscle viscoelasticity [57]. It can be emphasized that a model of the dynamics with precise parameters is not required in the inverse model.

The proposed forward model estimates the current limb state that is otherwise substantially delayed by the transmission along feedback loops. The model takes a copy of motor command and sensory information as inputs. The copy provides the forward model with information about the desired shape of the system dynamics designed in the inverse model. The forward model supposes that the inverse model realizes the desired dynamics during movements. Using the designed dynamics, the current limb state is estimated from the delayed sensory information through a recursive process. Note that a system model is not required in the proposed forward model.

The structure of the Smith predictor was used to enhance stability against feedback delays. In 1993, Miall and colleagues published a seminal study that showed the possibility that the forward model in dealing with feedback delays can be captured by the Smith predictor [35]. An estimate from the forward model is intentionally delayed to match the sensory delay and compared with the sensed state. The error between prediction and sensory feedback is then used to modulate the motor command. The forward model component in the Smith predictor, which requires a system model, is replaced with one that does not require a system model. More recently, Miall presented evidence against their Smith predictor model [58]. The study examined manual tracking tasks with a long visual feedback delay of 300 ms and found that adaptation to the delay led to reduction in tracking error and the mean power of tracking responses. However, the empirical results are against the prediction of an increase in response frequencies by the Smith predictor [58]. In the proposed model, it is assumed that the CNS keeps adjusting the values of the matrix $\bar{\beta }$ to improve performance as it adapts to the task. The matrix $\bar{\beta }$ has an effect on response frequencies, depending on its value. Hence, it would be possible to speculate that the proposed model is able to describe the empirical phenomena of the decrease in response frequencies in contrast to the Smith predictor. However, in this study the author focuses on the compensation of computational models for system dynamics, rather than the compensation for substantial delays. Here we do not look into the issue on whether or not the proposed model is capable of reproducing the tracking tasks with visual feedback delays.

### Neurophysiological correlates

How can the proposed computation be realized in a real biological system? The cerebellum receives afferent sensory information about the limb and reafference carrying copies of the motor commands and information required for movements including a desired trajectory from the primary motor, somatosensory and parietal cortex. Efferent copies of descending motor commands could be stored in the CNS and be transmitted by motor neurons [59]. Brodmann area 5 in the parietal cortex would be thought to possess a device that generates desired trajectories composed of position, velocity and acceleration values [4, 60, 61]. It was demonstrated that excitations of Brodmann area 5 cells were correlated with position, velocity and acceleration. Muscle length and lengthening velocity are measured through muscle spindles and mossy fibers [62]. The measured actual limb state ascends to the cerebral cortex and is inputted into the forward model after comparison. As for the states dealt with by the forward model, mossy fibers as well as area 5 cells are thought to transmit the acceleration component in addition to the position and velocity components [63]. The parietal lobe and cerebellum appear to play a crucial role in estimating the current state [64, 65].

### Single-joint fast movement

The ability of the proposed control to drive fast movements was evaluated. During fast movements for which duration is comparable to the delays of feedback loops, the roles of internal models in sensorimotor processing are emphasized [2, 53, 66]. Even during movements in which sensory information has an influence, online corrections depending only on sensory feedback could lead to instability. Feedback control is highly sensitive to delays and should maintain the open-loop gains low at high frequencies where the delays would introduce a phase lag of 180 degrees to prevent instability. This restriction makes feedback control impossible to produce fast movements.

In the simulation of the fast movement, it is observed that the optimal control model generates a trajectory following the minimum-jerk trajectory. The proposed control successfully reproduces the minimum-jerk trajectory. This indicates that the proposed forward model in the architecture of the Smith predictor efficiently estimates the current states of the limb and feeds them back to the inverse model. While the forward model in the optimal control generates the current states by simulating behaviors of the system using a model of the dynamics with precise parameters in the CNS, the proposed forward model calculates the current states from desired dynamics. In contrast, the equilibrium-point control model that does not involve internal models fails to reproduce the minimum-jerk movement, as shown in Fig 6.

### Two-joint fast movement

In the simulation of the multi-joint movement, it was investigated whether the proposed control compensates for system dynamics in multi-joint movements involving simultaneous motion at the shoulder and elbow. Interaction forces arise at one joint (e.g., the shoulder), because of motion of limb segments about other joints (e.g., the elbow), which include inertial forces from movements of other joints, centripetal torques and Coriolis torques, disturb achieving planned movements. That is, these interaction forces act as disturbances that need to be compensated. As movements become faster, interaction forces increase more. It was asserted that the cerebellum should possess a priori knowledge of the arm’s dynamics to compensate for interaction forces by simulating empirical movements with two kinds of computational models [4]. One of the computational models that is not equipped with an inverse model does not show accurate reproduction of fast movements, whereas the other one with an inverse model does. The proposed control does not show a notable difference in deviation between slow and fast movements, as shown in Fig 8. This suggests that the inverse model of the proposed control efficiently carries out dynamics compensation. Note that identifying interaction forces requires an explicit system model including the inertia and length of each segment of the limb (see Eq (19)). But the proposed control does not require a model of the dynamics with precise parameters. The proposed control builds inverse models using the relationship between the motor command and its responses. Through the compensation of the system dynamics and sensory delay, the proposed control captures the human’s voluntary movements, which is in agreement with a study presented in [67].

### Movement with unexpected inertial changes

The main characteristic of the proposed computational model is that it estimates and cancels out the system dynamics using a copy of motor outflow and (estimated) sensory inflow. This would have the system reach the target even when the properties of the system unexpectedly change. Model-based computations including the optimal control presented in this study probably show outcomes that depend on system properties. In the case of the optimal control, it could be that the forward model component generates an estimate or the inverse one produces a command based on the system model recognized in the CNS.

To investigate whether internal models in the CNS require a precise system model or not, an experimental study of [43] was revisited that evaluated the effect of changes in the inertial property of the controlled system on movement. They revealed that participants pointed to the target position regardless of whether an inertia was added or subtracted. Their second finding is that motor commands were designed and customized according to the inertia that the participants expected; EMG signals varied with the inertia to be presented. Another similar study showed that equifinality was preserved when the inertia of the arm system was changed unexpectedly [20]. The author would say that these studies not merely suppose that motor commands are tailored to the circumstance before the movements begin, but also imply that the movements might not be programmed using a model of the dynamics with precise parameters. From the view of the internal model hypothesis, equifinality has been refuted [12, 13, 68, 69]. Rather, equifinality is one of the characteristics supported by the equilibrium-point hypothesis that does not require a system model to calculate motor commends. However, the equilibrium-point hypothesis [18, 70–72], even with an assumption that it can deal with feedback delays, would be unable to explain the results by a study in [43]. Prediction by an equilibrium-point model would be similar to that by the optimal or proposed models during the ML condition, as shown in Fig 9. When the intermediate load is replaced with the high load and moved by an equilibrium-point controller; the commend designed for the intermediate load lacks to move the high load on the planned trajectory. But, in the case that the intermediate load is replaced with the low load, the system under equilibrium-point control would follow the trajectory that is displayed for the MM condition.

It would be possible to assume that the values of the matrix $\bar{\beta }$ are updated as the CNS adapts to a task. Indeed, Pinter and colleagues found that it takes 6-10 trial to adapt to a new inertial load [43]. It was also demonstrated that participants familiarized themselves with an inertial load through several trials [73]. After the customization trials, participants produce closely overlapping trajectories (perhaps the minimum-jerk trajectory) under the high and low loads (see Fig 1 in [43]). The proposed model produces the minimum-jerk trajectory if appropriate values of the gain $\bar{\beta }$ are found for the system with the high or low loads. If the value is much smaller or much larger than the one suitable to move the system along the planned trajectory, the produced command is correspondingly smaller or larger than the command that enables the system to stay on the planned trajectory during the movement. The value of the gain $\bar{\beta }$ impacts how accurately the system dynamics is estimated by TDE. TDE error, which is determined by the values of the matrix $\bar{\beta }$, leads to deviations from the planned trajectory; according to the value of the matrix $\bar{\beta }$, the movement can either fall short of or go beyond the planned trajectory during tracking. But, in the end, the movement approaches the planned end-point asymptotically. TDE error decreases as the movement begins to lose its momentum, diminishing the error between the planned position and actual position.

### Parameter sensitivity

**Fig 10:**
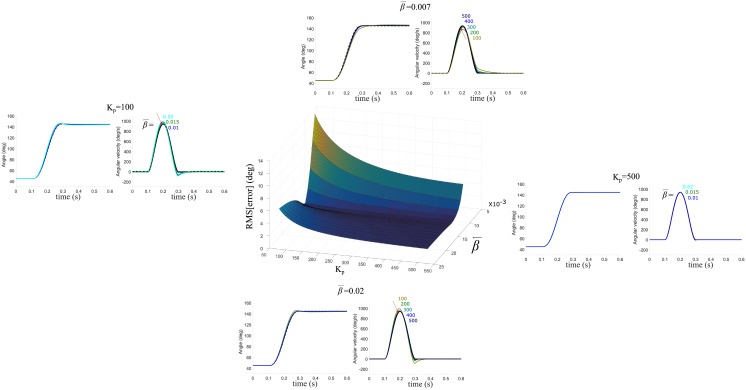
Sensitivity of the parameters $\bar{\beta }$ and *K*_*p*_. With $\bar{\beta }$ varying from 5×10^−3^ to 2×10^−2^ and *K*_*p*_ varying from 50 and 500, the root mean square error between the desired and actual trajectories during a single-joint fast movement is investigated. *K*_*v*_ is arbitrarily set as 0.4 times *K*_*p*_. Position and velocity profiles are presented for each case: (**Left**) the value of $\bar{\beta }$ varies while *K*_*p*_ is fixed at 100; (**Right**) the value of $\bar{\beta }$ varies while *K*_*p*_ is fixed at 500. (**Top**) the value of *K*_*p*_ varies between 100 and 500 with the value of $\bar{\beta }$ selected as 7×10^−3^; (**Bottom**) the value of *K*_*p*_ varies between 100 and 500 with the value of $\bar{\beta }$ selected as 2×10^−2^.

Fig 10 shows the sensitivity of the parameters $\bar{\beta }$ and *K*_*p*_ (*K*_*v*_ is arbitrarily set as 0.4*K*_*p*_) on tracking error between the desired and actual trajectories during a single-joint fast movement. Overall, it turns out that tracking error is low when $\bar{\beta }$ and *K*_*p*_ are both set high, whereas tracking performance becomes worse when $\bar{\beta }$ and *K*_*p*_ are both set low. The minimum-jerk trajectory is achieved in a satisfactory manner over wide ranges of the values of $\bar{\beta }$ and *K*_*p*_ (7×10^−3^ ≤ $\bar{\beta }$ and ≤ 2×10^−2^ 150 ≤ *K*_*p*_ ≤ 500). It is agreeable that fast movements are typically accompanied by high stiffness through muscle contractions and high stiffness would lead to accurate tracking. Meanwhile, the analysis shows that the limb fails to accurately track the desired trajectory even with high stiffness when $\bar{\beta }$ is set low (around 5×10^−3^ in this case), suggesting that dynamics compensation is poorly achieved. With relatively low muscle stiffness (around 50 in this case), both a low value and a great value of $\bar{\beta }$ lead to an increase in tracking error. These results perhaps originate from undercompensation and overcompensation of dynamics, while the stiffness is too low to suppress these compensation errors.

**Fig 11:**
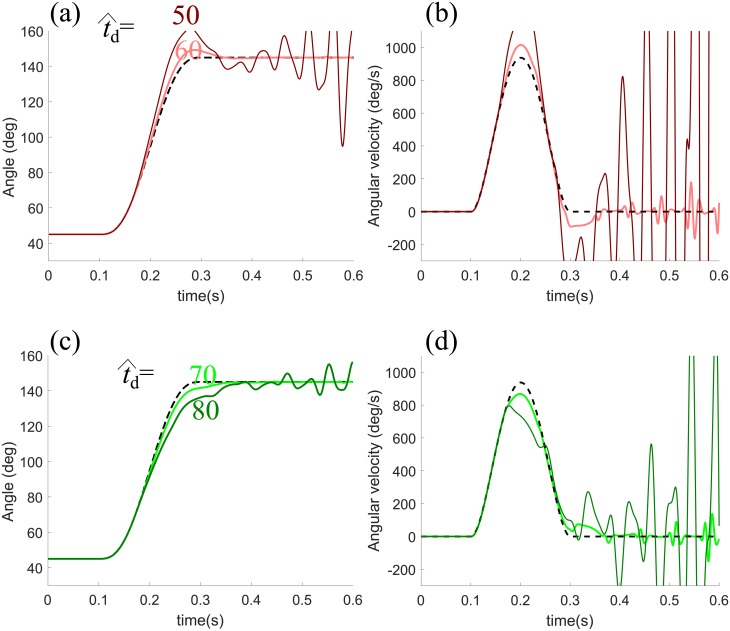
Sensitivity of the estimated delay ${\hat{t}}_{d}$. The effect of variations in the value of the estimated delay ${\hat{t}}_{d}$ is evaluated with the actual delay *t*_*d*_ anchored at 65 ms. The top profiles of position and velocity present the cases when the value of ${\hat{t}}_{d}$ is chosen as 50 and 60, which are both less than that of ${\hat{t}}_{d}$. The bottom profiles of position and velocity present the cases when the value of ${\hat{t}}_{d}$ is selected greater than that of ${\hat{t}}_{d}$ as 70 and 80.

Fig 11 displays the effect of the value of the estimated time delay ${\hat{t}}_{d}$ in feedback loops, while the value of the actual delay ${\hat{t}}_{d}$ is fixed at 65 ms. It is revealed that the proposed control model tolerates the difference in value between the actual delay and its estimate within a certain range regardless of whether or not the value of the estimate is greater than that the actual delay. However, the system is unstable with a great difference between the two values, which is in accordance with the study presented in [35].

## Appendix

### Role of matrix $\bar{\beta }$

The system (1) can be rewritten as

$$\begin{array}{c} M\left(\theta \right)\ddot{\theta }+f=\tau , \end{array}$$ (26)

where function variables are omitted for simplicity.

Eq (26) can also be re-expressed with a diagonal constant matrix $\bar{\beta }$, as

$$\begin{array}{c} \bar{\beta }\ddot{\theta }+H=\tau , \end{array}$$ (27)

where

$$\begin{array}{c} H≜\left(M\left(\theta \right)-\bar{\beta }\right)\ddot{\theta }+f. \end{array}$$ (28)

The control input (17) can be simplified as

$$\begin{array}{c} \tau_{\left(t\right)}=\bar{\beta }\nu_{\left(t\right)}+{\hat{H}}_{\left(t\right)}=\bar{\beta }\nu_{\left(t\right)}+H_{\left(t-dt\right)}, \end{array}$$ (29)

where $\nu ≜{\ddot{\theta }}_{r}+K_{v}\left({\dot{\theta }}_{r}-\dot{\hat{\theta }}\right)+K_{p}\left(\theta_{r}-\hat{\theta }\right).$

Originally, the proposed computational model compensates for *H*_(*t*)_ with its previous-step value *H*_(*t*−*dt*)_, but there exists a difference between *H*_(*t*)_ and *H*_(*t*−*dt*)_. The estimation error *ϵ* is defined as

$$\begin{array}{c} \epsilon_{\left(t\right)}≜{\bar{\beta }}^{-1}\left(H_{\left(t\right)}-H_{\left(t-dt\right)}\right). \end{array}$$ (30)

This estimation error can be written in terms of the new control input *ν* and the angular acceleration $\ddot{\theta }$ through a combination of Eqs (27) and (29) as

$$\begin{array}{c} \epsilon_{\left(t\right)}=\nu_{\left(t\right)}-{\ddot{\theta }}_{\left(t\right)}. \end{array}$$ (31)

From Eqs (26), (28), (29), and (31), a relationship can be drawn as follows:

$$\begin{array}{c} \begin{array}{cc} M\left(\theta_{\left(t\right)}\right)\epsilon_{\left(t\right)}= & M\left(\theta_{\left(t\right)}\right)\left(\nu_{\left(t\right)}-{\ddot{\theta }}_{\left(t\right)}\right)\\ = & M\left(\theta_{\left(t\right)}\right)\nu_{\left(t\right)}+f_{\left(t\right)}-\tau_{\left(t\right)}\\ = & M\left(\theta_{\left(t\right)}\right)\nu_{\left(t\right)}+f_{\left(t\right)}-\bar{\beta }\nu_{\left(t\right)}-H_{\left(t-dt\right)}\\ = & M\left(\theta_{\left(t\right)}\right)\nu_{\left(t\right)}+f_{\left(t\right)}-\bar{\beta }\nu_{\left(t\right)}-\left(M\left(\theta_{\left(t\right)}\right)-\bar{\beta }\right){\ddot{\theta }}_{\left(t-dt\right)}-f_{\left(t-dt\right)}\\ = & (M\left(\theta_{\left(t\right)}\right)-\bar{\beta })\nu_{\left(t\right)}-(M\left(\theta_{\left(t\right)}\right)-\bar{\beta }){\ddot{\theta }}_{\left(t-dt\right)}+f_{\left(t\right)}-f_{\left(t-dt\right)}\\ = & (M\left(\theta_{\left(t\right)}\right)-\bar{\beta })\nu_{\left(t\right)}-(M\left(\theta_{\left(t-dt\right)}\right)-\bar{\beta }){\ddot{\theta }}_{\left(t-dt\right)}\\ & -(M\left(\theta_{\left(t-dt\right)}\right)-M\left(\theta_{\left(t\right)}\right)){\ddot{\theta }}_{\left(t-dt\right)}+f_{\left(t\right)}-f_{\left(t-dt\right)}\\ = & (M\left(\theta_{\left(t\right)}\right)-\bar{\beta })\nu_{\left(t\right)}+(M\left(\theta_{\left(t-dt\right)}\right)-\bar{\beta })\left(\epsilon_{\left(t-dt\right)}-\nu_{\left(t-dt\right)}\right)\\ & -(M\left(\theta_{\left(t-dt\right)}\right)-M\left(\theta_{\left(t\right)}\right)){\ddot{\theta }}_{\left(t-dt\right)}+f_{\left(t\right)}-f_{\left(t-dt\right)}. \end{array} \end{array}$$ (32)

Multiplying the inverse of *M*(*θ*_(*t*)_) to the both hands of Eq (32) leads

$$\begin{array}{c} \epsilon_{\left(t\right)}=\left(I-M^{-1}\left(\theta_{\left(t\right)}\right)\bar{\beta }\right)\epsilon_{\left(t-dt\right)}+\left(I-M^{-1}\left(\theta_{\left(t\right)}\right)\bar{\beta }\right)\zeta_{\left(t-dt\right)}+\eta_{\left(t-dt\right)}, \end{array}$$ (33)

where

$$\begin{array}{c} \zeta_{\left(t-dt\right)}≜\nu_{\left(t\right)}-\nu_{\left(t-dt\right)}, \end{array}$$ (34)

$$\begin{array}{c} \eta_{\left(t-dt\right)}≜\left(I-M^{-1}\left(\theta_{\left(t\right)}\right)M\left(\theta_{\left(t-dt\right)}\right)\right){\ddot{\theta }}_{\left(t-dt\right)}+f_{\left(t\right)}-f_{\left(t-dt\right)}. \end{array}$$ (35)

Eq (33) is the closed-loop dynamics in terms of the estimation error *ϵ*.

These three equations can be rewritten in discrete-time domain as follows in order:

$$\begin{array}{c} \epsilon_{\left(k\right)}=\left(I-M^{-1}\left(\theta_{\left(k\right)}\right)\bar{\beta }\right)\epsilon_{\left(k-1\right)}+\left(I-M^{-1}\left(\theta_{\left(k\right)}\right)\bar{\beta }\right)\zeta_{\left(k-1\right)}+\eta_{\left(k-1\right)}, \end{array}$$ (36)

where

$$\begin{array}{c} \zeta_{\left(k-1\right)}≜\nu_{\left(k\right)}-\nu_{\left(k-1\right)}, \end{array}$$ (37)

$$\begin{array}{c} \eta_{\left(k-1\right)}≜\left(I-M^{-1}\left(\theta_{\left(k\right)}\right)M\left(\theta_{\left(k-1\right)}\right)\right){\ddot{\theta }}_{\left(k-1\right)}+f_{\left(k\right)}-f_{\left(k-1\right)}. \end{array}$$ (38)

From the viewpoint of the estimation error *ϵ*, the terms *η*, *ζ* in Eq (33) are regarded as forcing functions that are bounded in the case of a sufficiently small sampling period. If the roots of $\left(I-M^{-1}\left(\theta_{\left(k\right)}\right)\bar{\beta }\right)$ are all placed inside the unit circle, *ϵ* is asymptotically bounded [74]. The coefficient $(\left(I-M^{-1}\left(\theta_{\left(k\right)}\right)\bar{\beta }\right)$, in particular, the matrix $\bar{\beta }$ determines how accurately dynamics estimation is achieved; the gains affect the difference between *H* and $\hat{H}$, which eventually affect the tracking error [75].

### Optimal control

The control law of optimal control can be typically designed as

$$\begin{array}{c} \tau =K[\begin{array}{c} q_{d}-\hat{q}\\ {\dot{q}}_{d}-\dot{\hat{q}} \end{array}], \end{array}$$ (39)

where *K* denotes a matrix.

The state feedback gain matrix *K* is selected in a way to minimize the following performance index:

$$\begin{array}{c} I=\int_{0}^{\infty }e^{T}Qe+\tau^{T}R\tau dt, \end{array}$$ (40)

where $e≜{\left[{\left(q_{d}-\hat{q}\right)}^{T}\;{\left({\dot{q}}_{d}-\dot{\hat{q}}\right)}^{T}\right]}^{T}$, and *Q* denotes an a state-weighting matrix and *R* denotes an input-weighting matrix. Note that these methods require a model of the dynamics with precise parameters to determine *K* that minimizes the index *I*.

Estimates of the current states required for motor command are provided by a predictor that acts as the forward model in the CNS, which can be constructed as [47]

$$\begin{array}{c} {}[\begin{array}{c} \hat{q}\\ \dot{\hat{q}} \end{array}]=e^{A_{s}{\hat{t}}_{d}}[\begin{array}{c} q(t-t_{d})\\ \dot{q}\left(t-t_{d}\right) \end{array}]+\int_{0}^{{\hat{t}}_{d}}e^{A_{s}t^{\prime }}B_{s}\tau \left(t-t^{\prime }\right)dt^{\prime }, \end{array}$$ (41)

where *A*_*s*_ and *B*_*s*_ are matrices, respectively.

The predictor estimates the current states from the sensed states, which are *t*_*d*_-ms delayed, based on a system model (matrices *A*_*s*_ and *B*_*s*_). The matrices *A*_*s*_ and *B*_*s*_ represents the limb system in state space:

$$\begin{array}{c} {}[\begin{array}{c} \dot{q}\\ \ddot{q} \end{array}]=A_{s}[\begin{array}{c} q\\ \dot{q} \end{array}]+B_{s}\tau . \end{array}$$ (42)

In the case of the forearm, the matrices *A*_*s*_ and *B*_*s*_ can be defined as, neglecting viscoelasticity,

$$\begin{array}{c} A_{s}=[\begin{array}{cc} 0 & 1\\ 0 & 0 \end{array}],\;B_{s}=1/J_{2}. \end{array}$$ (43)

## References

[1] Wolpert DM, Ghahramani Z, Jordan MI. An internal model for sensorimotor integration. Science-AAAS-Weekly Paper Edition. 1995;269(5232):1880–1882. 10.1126/science.7569931 7569931

[2] Miall RC, Wolpert DM. Forward models for physiological motor control. Neural networks. 1996;9(8):1265–1279. 10.1016/S0893-6080(96)00035-4 12662535

[3] Wolpert DM, Miall RC, Kawato M. Internal models in the cerebellum. Trends in cognitive sciences. 1998;2(9):338–347. 10.1016/S1364-6613(98)01221-2 21227230

[4] Schweighofer N, Arbib MA, Kawato M. Role of the cerebellum in reaching movements in humans. I. Distributed inverse dynamics control. European Journal of Neuroscience. 1998;10(1):86–94. 10.1046/j.1460-9568.1998.00006.x 9753116

[5] Kawato M. Internal models for motor control and trajectory planning. Current opinion in neurobiology. 1999;9(6):718–727. 10.1016/S0959-4388(99)00028-8 10607637

[6] Wolpert DM, Ghahramani Z. Computational principles of movement neuroscience. nature neuroscience. 2000;3:1212–1217. 10.1038/81497 11127840

[7] Dingwell JB, Mah CD, Mussa-Ivaldi FA. Manipulating objects with internal degrees of freedom: evidence for model-based control. Journal of Neurophysiology. 2002;88(1):222–235. 10.1152/jn.2002.88.1.222 12091548

[8] Kawato M, Kuroda T, Imamizu H, Nakano E, Miyauchi S, Yoshioka T. Internal forward models in the cerebellum: fMRI study on grip force and load force coupling In: Progress in brain research. vol. 142 Elsevier; 2003 p. 171–188. 12693261 10.1016/S0079-6123(03)42013-X

[9] Kurtzer IL, Pruszynski JA, Scott SH. Long-latency reflexes of the human arm reflect an internal model of limb dynamics. Current Biology. 2008;18(6):449–453. 10.1016/j.cub.2008.02.053 18356051

[10] Popa LS, Hewitt AL, Ebner TJ. Purkinje cell simple spike discharge encodes error signals consistent with a forward internal model. The Cerebellum. 2013;12(3):331–333. 10.1007/s12311-013-0452-4 23361619 PMC3643991

[11] Shadmehr R, Mussa-Ivaldi FA. Adaptive representation of dynamics during learning of a motor task. The Journal of Neuroscience. 1994;14(5):3208–3224. 10.1523/JNEUROSCI.14-05-03208.1994 8182467 PMC6577492

[12] Lackner JR, Dizio P. Rapid adaptation to Coriolis force perturbations of arm trajectory. Journal of neurophysiology. 1994;72(1):299–313. 10.1152/jn.1994.72.1.299 7965013

[13] Hinder MR, Milner TE. The case for an internal dynamics model versus equilibrium point control in human movement. The Journal of Physiology. 2003;549(3):953–963. 10.1113/jphysiol.2002.033845 12717002 PMC2342993

[14] Cluff T, Scott SH. Rapid feedback responses correlate with reach adaptation and properties of novel upper limb loads. Journal of Neuroscience. 2013;33(40):15903–15914. 10.1523/JNEUROSCI.0263-13.2013 24089496 PMC6618484

[15] Shadmehr R. Learning to predict and control the physics of our movements. Journal of neuroscience. 2017;37(7):1663–1671. 10.1523/JNEUROSCI.1675-16.2016 28202784 PMC5320601

[16] Rothwell J, Traub M, Marsden C. Automatic and ‘voluntary’responses compensating for disturbances of human thumb movements. Brain research. 1982;248(1):33–41. 10.1016/0006-8993(82)91144-1 7127140

[17] Jaric S, Corcos DM, Gottlieb GL, Ilic DB, Latash ML. The effects of practice on movement distance and final position reproduction: implications for the equilibrium-point control of movements. Experimental Brain Research. 1994;100(2):353–359. 10.1007/BF00227205 7813672

[18] Feldman AG, Levin MF. The origin and use of positional frames of reference in motor control. Behavioral and Brain Sciences. 1995;18(04):723–744. 10.1017/S0140525X0004070X

[19] Ilic D, Corcos DM, Gottlieb GL, Latash ML, Jaric S. The effects of practice on movement reproduction: Implications for models of motor control. Human movement science. 1996;15(1):101–114. 10.1016/0167-9457(95)00042-9

[20] Jaric S, Milanovic S, Blesic S, Latash ML. Changes in movement kinematics during single-joint movements against expectedly and unexpectedly changed inertial loads. Human movement science. 1999;18(1):49–66. 10.1016/S0167-9457(98)00033-5

[21] Feldman AG, Latash ML. Testing hypotheses and the advancement of science: recent attempts to falsify the equilibrium point hypothesis. Experimental Brain Research. 2005;161(1):91–103. 10.1007/s00221-004-2049-0 15490137

[22] Bizzi E, Accornero N, Chapple W, Hogan N. Posture control and trajectory formation during arm movement. Journal of Neuroscience. 1984;4(11):2738–2744. 10.1523/JNEUROSCI.04-11-02738.1984 6502202 PMC6564726

[23] Hwang EJ, Smith MA, Shadmehr R. Adaptation and generalization in acceleration-dependent force fields. Experimental brain research. 2006;169(4):496–506. 10.1007/s00221-005-0163-2 16292640 PMC1456064

[24] Burdet E, Tee KP, Mareels I, Milner TE, Chew CM, Franklin DW, et al Stability and motor adaptation in human arm movements. Biological cybernetics. 2006;94(1):20–32. 10.1007/s00422-005-0025-9 16283374

[25] Van Ooteghem K, Frank J, Allard F, Buchanan J, Oates A, Horak F. Compensatory postural adaptations during continuous, variable amplitude perturbations reveal generalized rather than sequence-specific learning. Experimental brain research. 2008;187(4):603–611. 10.1007/s00221-008-1329-5 18327574 PMC2855617

[26] Schmid M, Bottaro A, Sozzi S, Schieppati M. Adaptation to continuous perturbation of balance: progressive reduction of postural muscle activity with invariant or increasing oscillations of the center of mass depending on perturbation frequency and vision conditions. Human movement science. 2011;30(2):262–278. 10.1016/j.humov.2011.02.002 21440318

[27] Fortney K, Tweed DB. Computational advantages of reverberating loops for sensorimotor learning. Neural computation. 2012;24(3):611–634. 10.1162/NECO_a_00237 22091669

[28] Crevecoeur F, Thonanrd JL, Lefevre P. A sub-movement time scale of human motor adaptation. bioRxiv. 2018. 10.1523/ENEURO.0149-19.2019 PMC7004489 31949026

[29] Youcef-Toumi K, Ito O. A time delay controller for systems with unknown dynamics. Journal of dynamic systems, measurement, and control. 1990;112(1):133–142. 10.1115/1.2894130

[30] Hsia TS, Lasky T, Guo Z. Robust independent joint controller design for industrial robot manipulators. Industrial Electronics, IEEE Transactions on. 1991;38(1):21–25. 10.1109/41.103479

[31] Chang PH, Jeong JW. Enhanced operational space formulation for multiple tasks by using time-delay estimation. Robotics, IEEE Transactions on. 2012;28(4):773–786. 10.1109/TRO.2012.2187397

[32] Kim D, Gillespie RB, Chang PH. Simple, robust control and synchronization of the Lorenz system. Nonlinear Dynamics. 2013; p. 1–10.

[33] Baek J, Jin M, Han S. A new adaptive sliding-mode control scheme for application to robot manipulators. IEEE Transactions on Industrial Electronics. 2016;63(6):3628–3637. 10.1109/TIE.2016.2522386

[34] Kim S, Bae J. Force-mode control of rotary series elastic actuators in a lower extremity exoskeleton using model-inverse time delay control (MiTDC). IEEE/ASME Transactions on Mechatronics. 2017;.

[35] Miall R, Weir D, Wolpert DM, Stein J. Is the cerebellum a smith predictor? Journal of motor behavior. 1993;25(3):203–216. 10.1080/00222895.1993.9942050 12581990

[36] De Rugy A, Loeb GE, Carroll TJ. Muscle coordination is habitual rather than optimal. Journal of Neuroscience. 2012;32(21):7384–7391. 10.1523/JNEUROSCI.5792-11.2012 22623684 PMC6622296

[37] Lee BC, Thrasher TA, Layne CS, Martin BJ. Vibrotactile cuing revisited to reveal a possible challenge to sensorimotor adaptation. Experimental brain research. 2016;234(12):3523–3530. 10.1007/s00221-016-4750-1 27501732

[38] Jagacinski RJ, Flach JM. Control theory for humans: Quantitative approaches to modeling performance. CRC Press; 2002.

[39] Smith OJ. A controller to overcome dead time. ISA Journal. 1959;6(2):28–33.

[40] Flash T, Hogan N. The coordination of arm movements: an experimentally confirmed mathematical model. The journal of Neuroscience. 1985;5(7):1688–1703. 10.1523/JNEUROSCI.05-07-01688.1985 4020415 PMC6565116

[41] Kistemaker DA, Van Soest AKJ, Bobbert MF. Is equilibrium point control feasible for fast goal-directed single-joint movements? Journal of Neurophysiology. 2006;95(5):2898–2912. 10.1152/jn.00983.2005 16436480

[42] Koike Y, Kawato M. Estimation of dynamic joint torques and trajectory formation from surface electromyography signals using a neural network model. Biological cybernetics. 1995;73(4):291–300. 10.1007/BF00199465 7578470

[43] Pinter IJ, Bobbert MF, Smeets JB, et al Motor commands for fast point-to-point arm movements are customized for small changes in inertial load. Journal of Electromyography and Kinesiology. 2011;21(6):960–967. 10.1016/j.jelekin.2011.08.001 21890379

[44] Kleinman D. Optimal control of linear systems with time-delay and observation noise. IEEE Transactions on Automatic Control. 1969;14(5):524–527. 10.1109/TAC.1969.1099242

[45] Todorov E, Jordan MI. Optimal feedback control as a theory of motor coordination. Nature neuroscience. 2002;5(11):1226–1235. 10.1038/nn963 12404008

[46] Diedrichsen J, Shadmehr R, Ivry RB. The coordination of movement: optimal feedback control and beyond. Trends in cognitive sciences. 2010;14(1):31–39. 10.1016/j.tics.2009.11.004 20005767 PMC4350769

[47] Gawthrop P, Loram I, Lakie M, Gollee H. Intermittent control: a computational theory of human control. Biological cybernetics. 2011;104(1-2):31–51. 10.1007/s00422-010-0416-4 21327829

[48] McIntyre J, Bizzi E. Servo hypotheses for the biological control of movement. Journal of motor behavior. 1993;25(3):193–202. 10.1080/00222895.1993.9942049 12581989

[49] de Lussanet MH, Smeets JB, Brenner E. Relative damping improves linear mass-spring models of goal-directed movements. Human movement science. 2002;21(1):85–100. 10.1016/S0167-9457(02)00075-1 11983435

[50] Popescu FC, Rymer WZ. Implications of Low Mechanical Impedence in Upper Limb Reaching Motion. MOTOR CONTROL-CHAMPAIGN-. 2003;7(4):323–327. 10.1123/mcj.7.4.323 14999131

[51] Shadmehr R, Krakauer JW. A computational neuroanatomy for motor control. Experimental Brain Research. 2008;185(3):359–381. 10.1007/s00221-008-1280-5 18251019 PMC2553854

[52] Friston K. What is optimal about motor control? Neuron. 2011;72(3):488–498. 10.1016/j.neuron.2011.10.018 22078508

[53] Crevecoeur F, Scott SH. Beyond muscles stiffness: importance of state-estimation to account for very fast motor corrections. PLoS computational biology. 2014;10(10):e1003869 10.1371/journal.pcbi.1003869 25299461 PMC4191878

[54] Hoff B, Arbib MA. Models of trajectory formation and temporal interaction of reach and grasp. Journal of motor behavior. 1993;25(3):175–192. 10.1080/00222895.1993.9942048 12581988

[55] Desmurget M, Rossetti Y, Prablanc C, Jeannerod M, Stelmach GE. Representation of hand position prior to movement and motor variability. Canadian journal of physiology and pharmacology. 1995;73(2):262–272. 10.1139/y95-037 7621365

[56] Bard C, Turrell Y, Fleury M, Teasdale N, Lamarre Y, Martin O. Deafferentation and pointing with visual double-step perturbations. Experimental brain research. 1999;125(4):410–416. 10.1007/s002210050697 10323286

[57] van Soest AJ, Bobbert MF. The contribution of muscle properties in the control of explosive movements. Biological cybernetics. 1993;69(3):195–204. 10.1007/BF00198959 8373890

[58] Miall R, Jackson J. Adaptation to visual feedback delays in manual tracking: evidence against the Smith Predictor model of human visually guided action. Experimental Brain Research. 2006;172(1):77–84. 10.1007/s00221-005-0306-5 16424978

[59] Holst E, Mittelstaedt H. Das reafferenzprinzip. Naturwissenschaften. 1950;37(20):464–476. 10.1007/BF00622503

[60] Kalaska J, Cohen D, Prud’Homme M, Hyde M. Parietal area 5 neuronal activity encodes movement kinematics, not movement dynamics. Experimental Brain Research. 1990;80(2):351–364. 10.1007/BF00228162 2113482

[61] Battaglia-Mayer A, Caminiti R, Lacquaniti F, Zago M. Multiple levels of representation of reaching in the parieto-frontal network. Cerebral Cortex. 2003;13(10):1009–1022. 10.1093/cercor/13.10.1009 12967918

[62] Van Kan PL, Houk JC, Gibson AR. Output organization of intermediate cerebellum of the monkey. Journal of neurophysiology. 1993;69(1):57–73. 10.1152/jn.1993.69.1.57 8433134

[63] Van Kan PL, Gibson AR, Houk JC. Movement-related inputs to intermediate cerebellum of the monkey. Journal of neurophysiology. 1993;69(1):74–94. 10.1152/jn.1993.69.1.74 8433135

[64] Eskandar EN, Assad JA. Dissociation of visual, motor and predictive signals in parietal cortex during visual guidance. Nature neuroscience. 1999;2(1):88–93. 10.1038/4594 10195185

[65] Desmurget M, Epstein C, Turner R, Prablanc C, Alexander G, Grafton S. Role of the posterior parietal cortex in updating reaching movements to a visual target. Nature neuroscience. 1999;2(6):563–567. 10.1038/9219 10448222

[66] Desmurget M, Grafton S. Forward modeling allows feedback control for fast reaching movements. Trends in cognitive sciences. 2000;4(11):423–431. 10.1016/S1364-6613(00)01537-0 11058820

[67] Gritsenko V, Yakovenko S, Kalaska JF. Integration of predictive feedforward and sensory feedback signals for online control of visually guided movement. Journal of Neurophysiology. 2009;102(2):914–930. 10.1152/jn.91324.2008 19474166

[68] Popescu FC, Rymer WZ. End points of planar reaching movements are disrupted by small force pulses: an evaluation of the hypothesis of equifinality. Journal of Neurophysiology. 2000;84(5):2670–2679. 10.1152/jn.2000.84.5.2670 11068008

[69] Zhou T, Solnik S, Wu YH, Latash ML. Unintentional movements produced by back-coupling between the actual and referent body configurations: violations of equifinality in multi-joint positional tasks. Experimental brain research. 2014;232(12):3847–3859. 10.1007/s00221-014-4059-x 25150552 PMC4241166

[70] Feldman AG. Once more on the equilibrium-point hypothesis (λ model) for motor control. Journal of motor behavior. 1986;18(1):17–54. 10.1080/00222895.1986.10735369 15136283

[71] Feldman AG, Levin MF. The equilibrium-point hypothesis–past, present and future In: Progress in motor control. Springer; 2009 p. 699–726. 10.1007/978-0-387-77064-2_38 19227529

[72] Latash M. Evolution of motor control: from reflexes and motor programs to the equilibrium-point hypothesis. Journal of human kinetics. 2008;19:3–24. 10.2478/v10078-008-0001-2 19823595 PMC2759721

[73] Krakauer JW, Ghilardi MF, Ghez C. Independent learning of internal models for kinematic and dynamic control of reaching. Nature neuroscience. 1999;2(11):1026–1031. 10.1038/14826 10526344

[74] Jin M, Lee J, Chang PH, Choi C. Practical nonsingular terminal sliding-mode control of robot manipulators for high-accuracy tracking control. IEEE Transactions on Industrial Electronics. 2009;56(9):3593–3601. 10.1109/TIE.2009.2024097

[75] Hsia T, Gao L. Robot manipulator control using decentralized linear time-invariant time-delayed joint controllers. In: Robotics and Automation, 1990. Proceedings., 1990 IEEE International Conference on. IEEE; 1990. p. 2070–2075.

